# A Longitudinal Study of Premalignant Gastric Lesions and Early Onset Gastric Cancer Among Young Adults in Central Saudi Arabia

**DOI:** 10.3390/curroncol32080428

**Published:** 2025-07-30

**Authors:** Ahmed Albadrani, Georgios Zacharakis, Mohammed Saad Alqahtani, Abdulrahman AlHarbi, Abdulaziz Alkudam, Abdullah Bawazir, Naif Albulayhid, Majed Zaki Bahader, Ahmed Mohammed Alghayyamah, Zahraa Saeed Alzaher

**Affiliations:** 1 Division of Gastroenterology, Department of Internal Medicine, College of Medicine, Prince Sattam bin Abdulaziz University Hospital, Prince Sattam bin Abdulaziz University, Al Kharj 11942, Saudi Arabia; a.albadrani@psau.edu.sa (A.A.); abdulrahmanha03@gmail.com (A.A.); al.kadam@hotmail.com (A.A.); abawazir@kfmc.med.sa (A.B.); nalbulayhid@gmail.com (N.A.); 2 Endoscopy Unit, Department of Internal Medicine, Arryan Hospital, Dr. Sulaiman Al Habib Ar Rayyan Hospital, Riyadh 14212, Saudi Arabia; majed.bahader@hmg.local (M.Z.B.); amalghayyamah@kaauh.edu.sa (A.M.A.); zahraa.alzaher@hmg.local (Z.S.A.); 3 Department of Internal Medicine, College of Medicine, Prince Sattam bin Abdulaziz University, Al Kharj 11942, Saudi Arabia; ms.alqahtani@psau.edu.sa; 4 Department of Internal Medicine, King Fahad Medical City, Riyadh 12231, Saudi Arabia

**Keywords:** early onset gastric cancer, precancerous gastric lesions, prospective study, in young adults

## Abstract

Gastric cancer traditionally affects older adults, and its precursor lesions and risk factors are well-documented in this population. *Helicobacter pylori* (*H. pylori*) infection remains highly prevalent in Saudi Arabia and contributes to gastric pathology. However, early-onset gastric cancer (EOGC), diagnosed in individuals aged ≤ 45 years, presents unique challenges and remains poorly understood in young populations. Therefore, we conducted an observational cohort study using a prospective longitudinal design (2021–2024) involving 1823 Saudi nationals aged 18–45 years who underwent zoom high-definition chromoendoscopy to evaluate the prevalence of premalignant gastric lesions (PGLs) and EOGC. We found a high *H. pylori* prevalence (78.0%) with PGLs in 1.9% of participants and EOGC-adenocarcinoma in 0.7% of patients. All EOGC cases arose from dysplasia, with most PGLs being classified as OLGA/OLGIM stage II/III. Multiple risk factorswere significantly associated with PGLs and EOGC, including *H. pylori* infection (*p* = 0.022), increasing age (*p* < 0.001), a family history of gastric cancer (*p* < 0.001), poor dietary habits (*p* < 0.001), obesity (*p* < 0.001), and smoking (*p* < 0.001). Additional EOGC risk factors include dage of 36–45 years (*p* = 0.018), EBV infection (*p* = 0.016), and diabetes mellitus (*p* = 0.001). These findings demonstrate the notable presence of PGLs and EOGC in young Saudi adults and emphasize the importance of early detection and risk factor management in this vulnerable population.

## Simple Summary

We aimed to shed light on the prevalence and risk factors of early-onset gastric cancer (EOGC) and premalignant gastric lesions (PGLs) among young Saudi adults, a group traditionally considered to be at lower risk and underrepresented in gastric cancer research. By conducting a large-scale, prospective cohort study using zoom high-definition chromoendoscopy, the authors provided valuable epidemiological data that challenged existing age-related assumptions about gastric cancer. Their findings highlight the significant role of *Helicobacter pylori*, lifestyle factors, and comorbidities such as diabetes in EOGC development. This research may significantly impact medical and research communities by prompting earlier screening guidelines, informing targeted prevention strategies, and encouraging further investigation into region-specific risk factors.

## 1. Introduction

Gastric cancer (GC) diagnosed in individuals aged 45 years or younger is often defined as EOGC, although some studies have used thresholds of 40 or 50 [1]. The underlying pathogenesis of EOGC remains unclear, but it accounts for a notable proportion, estimated at around 10%, of all GC cases globally [1]. In 2020, a significant number of new EOGC cases were reported worldwide, with varying incidence rates across regions; lower rates have been observed in Europe and the USA compared to higher rates in China and South Korea [1,2,3].

While the overall incidence of GC shows a decline in many areas, the trends for EOGC among young individuals (<45 years) fluctuate, with reports of decreasing, stable, or increasing rates in different countries, including the USA [4,5,6], Brazil, Denmark, India, Israel, Korea [3], and The Netherlands [7]. In Saudi Arabia, data gathered from the Saudi Cancer Registry for the period 2006–2016 indicated that late-onset GC (LOGC) was most common in individuals aged >75 years, while EOGC rates were lowest among individuals aged 0–19 years [8]. Projections based on global trends suggest potential future increases in EOGC incidence in some high-risk countries, while rates might stabilize or decline elsewhere [9]. Despite these observations, identifying populations at high risk of EOGC remains a global challenge owing to limited epidemiological data [1,9].

Risk factors for EOGC may differ significantly from those established for LOGC [1,10]. While *Helicobacter pylori* (*H. pylori*) infection is a primary driver for both [1,11], its role in EOGC might involve the early acquisition of virulent strains rather than the long-term chronic atrophy typically preceding LOGC [1]. Genetic predisposition and family history are considered particularly relevant for EOGC identification, along with histological findings from PGLs according to international guidelines [1,12,13,14,15,16]. This contrasts with traditional LOGC risk factors such as advanced age (>50 years), male sex, and potentially autoimmune conditions such as pernicious anemia, which are less common in EOGC screening populations [12,13,14,15,16]. Furthermore, lifestyle factors pertinent to younger generations may disproportionately influence EOGC risk; these include shifts towards “Western” dietary patterns (e.g., higher processed meat intake and lower fruit/vegetable intake) [1,11] and high salt intake (traditionally linked more to LOGC) [17]. Alcohol consumption, a known risk factor globally [18,19], is not applicable in Saudi Arabia because of legal and cultural prohibitions. Smoking is also a well-established risk factor, linked to the general trend of smoking initiation at an early age [19,20]. Exploring these differing risk profiles is crucial for understanding EOGC [10].

PGLs, including atrophic gastritis (AG), gastric intestinal metaplasia (GIM), and dysplasia, are key steps in the Correa cascade, a stepwise progression from chronic inflammation to intestinal-type GC, and are strongly associated with *H. pylori* infection [12,15]. Another important course is autoimmune atrophic gastritis [12,15]. A recent systematic review reported the global adult prevalence of AG, GIM, and dysplasia, but the data were not stratified by young age groups [21]. Generally, PGL prevalence is lower in younger individuals than in older adults [22,23,24,25], although some studies have suggested trends, such as an increasing prevalence of AG among adults aged 35–44 years in certain populations [26]. Given that PGLs can progress to cancer, understanding their prevalence in the EOGC age group is important [21].

Saudi Arabia is considered a low-incidence country for GC overall [27,28]; however, it reports a high prevalence of *H. pylori* infection, which varies regionally [29]. Limited data exist on the association between environmental risk factors and GC in this setting [29]. The high prevalence of *H. pylori* among young Saudis is a significant factor potentially contributing to the development of PGLs [30,31]. Therefore, evaluating the burden of PGLs and EOGC in this population is crucial.

This study aimed to evaluate the prevalence of PGLs (chronic *H. pylori* gastritis, AG, GIM, and dysplasia) and EOGC in a cohort of symptomatic young adults (18–45 years) in central Saudi Arabia. We also sought to identify potential risk factors associated with these conditions in this population, including *H. pylori* status, demographic factors, lifestyle habits (smoking, BMI, and diet), and relevant comorbidities such as diabetes mellitus.

## 2. Methods

### 2.1. Study Design and Setting

This was an observational cohort study with a longitudinal design. conducted between 2021 and 2024 in central Saudi Arabia. This study screened young Saudi adults presenting with upper gastrointestinal (GI) symptoms who underwent outpatient upper GI endoscopy. The participating centers included the Endoscopy Unit of Prince Sattam bin Abdulaziz University Hospital (Al Kharj, Saudi Arabia), Arryan Hospital, Dr. Sulaiman Al Habib Medical Services Group (Riyadh, Saudi Arabia), and King Fahad Medical City (Riyadh, Saudi Arabia).

### 2.2. Participants

Eligible participants were Saudi nationals aged 18 to 45 years who presented with upper GI symptoms necessitating upper endoscopy. The exclusion criteria included prior GC or PGL diagnoses, a history of *H. pylori* infection, comorbidities contraindicating endoscopy (e.g., advanced portal hypertension), conditions likely explaining symptoms (e.g., severe gastroesophageal reflux disease managed with specific protocols, dysphagia, or persistent vomiting suggestive of alternative diagnoses), significant unexplained weight loss or anemia requiring urgent inpatient evaluation, and previous gastric surgery.

### 2.3. Data Collection

Demographic information (age, sex, and region of residence), family history of cancer, body mass index (BMI), smoking status, and dietary habits (consumption of a high-salt diet, red/processed meats, vegetables, and fruits) were collected using a standardized questionnaire (Supplementary Materials). Clinical data included the presence of diabetes mellitus (DM) and autoimmune conditions, specifically pernicious anemia.

### 2.4. Endoscopic Procedure and Biopsy Protocol

Upper GI endoscopy was performed using zoom (magnification) high-definition endoscopes equipped with virtual chromoendoscopy (HD-CE, e.g., Narrow-Band Imaging [NBI, Olympus, Tokyo, Japan] or i-scan [Pentax, Tokyo, Japan]) and magnification/zoom capabilities. The gastric mucosa was systematically evaluated to identify *H. pylori* infection, PGLs (AG, GIM, and dysplasia), and EOGC in accordance with international guidelines [12,13,16]. Endoscopic findings suggestive of PGLs or neoplasia were documented using the VS classification system for magnified views [32,33,34].

Targeted biopsies were obtained from suspicious lesions that were identified during HD-CE. Additionally, mapping biopsies were taken according to the updated Sydney System protocol and a minimum of five non-targeted biopsies (two from the antrum, two from the corpus, and one from the incisura angularis) were collected in separate, labeled containers to assess background gastritis, atrophy, and metaplasia [35]. For patients requiring sedation for procedure completion, propofol was administered.

### 2.5. Histopathology and Ancillary Testing

Biopsy specimens were fixed in 10% neutral buffered formalin. A single experienced pathologist, blinded to the clinical details beyond the biopsy site, examined all tissue samples using hematoxylin–eosin (H&E) staining for *H. pylori* detection. Chronic gastritis grading followed the updated Sydney System criteria [35]. Histological staging of atrophy and GIM was performed using the Operative Link on Gastritis Assessment (OLGA) and/or Operative Link on Gastric Intestinal Metaplasia Assessment (OLGIM) systems for risk stratification [36,37,38]. Dysplasia was classified according to standard criteria. *H. pylori* status was confirmed histologically based on H&E stains [39]. Epstein–Barr virus (EBV) presence was assessed using in situ hybridization for EBERs and immunohistochemistry [40]. Histological micrographs were not taken to confirm endoscopic lesions because standard clinical practice does not always require imaging. In our clinical study histopathological reports were considered sufficient for diagnosis. There was no specialized equipment such as microscope cameras or trained personnel in any of the hospitals.

### 2.6. Treatment of HP Infection

Upon histological confirmation of *H. pylori* infection, confirmed for the first time in patients undergoing upper GI endoscopy, we treated these patients for *H. pylori* eradication. We followed the practice guidelines for the management of *Helicobacter pylori* infection provided by the Saudi *H. pylori* Working Group [29]. The exact regimens have been described previously [31]. *H. pylori* eradication was assessed using the urea breath test and stool, or the stool *H. pylori* antigen test after 1 month of *H. pylori* treatment [29].

### 2.7. Laboratory Investigations

In patients with histological findings suggestive of autoimmune atrophic gastritis, pernicious anemia was investigated by testing for serum anti-intrinsic factor (anti-IF) and anti-parietal cell antibodies (APCA). Serum chromogranin A and gastrin levels were measured if neuroendocrine neoplasms were suspected based on endoscopic or histological findings.

### 2.8. Statistical Analysis

All statistical analyses were performed using IBM SPSS Statistics for Windows, Version 26.0 (IBM Corp., Armonk, NY, USA). Descriptive statistics were used to summarize participant characteristics with mean (standard deviation—SD)for continuous variables and number (percent) for categorical values. Group comparisons were performed using chi-squared tests. Statistical significance was set at *p* < 0.05. Binary logistic regression analysis was performed to identify possible factors associated with PGLs and EOGC in young adults. *H. pylori* status, EBV, demographic factors, lifestyle habits (smoking, BMI, and diet), and relevant comorbidities such as diabetes mellitus were used as independent variables in binary logistic regression.

## 3. Results

**Figure 1:**
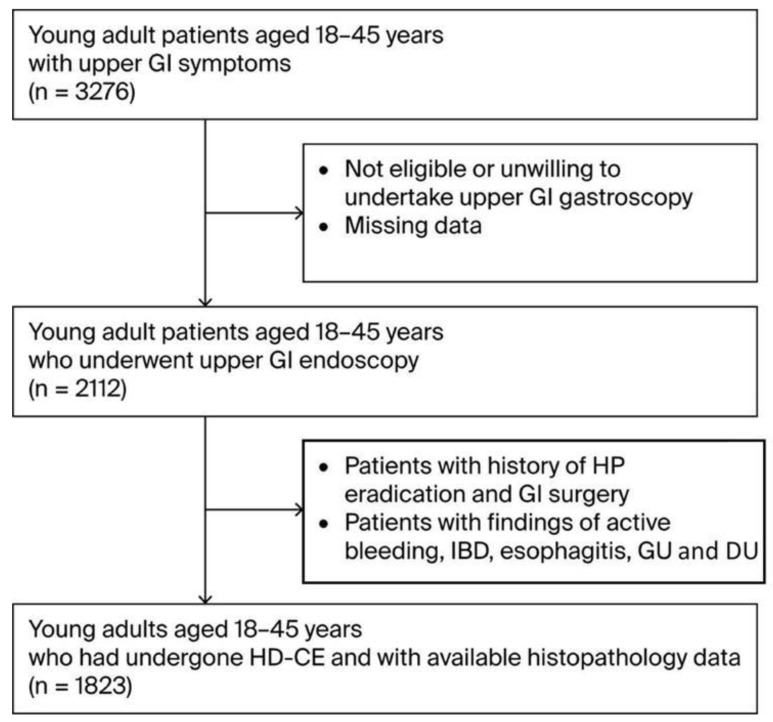
Flow diagram of patient selection. Abbreviations: GI—gastrointestinal; IBD—Inflammatory Bowel Disease; GU—Gastric Ulcer; DU—Duodenal Ulcer.

During the three-year study period (2021–2024), 1823 participants aged 18–45 years who presented with upper GI symptoms were enrolled in the study after meeting the eligibility criteria (Figure 1). The demographic and baseline characteristics of this cohort are presented in Table 1. The mean age of the participants was 36.0 (±11.3) years, with the cohort compromising 981 males (53.8%) and 842 females (46.2%). Participants were divided into three age groups: 21% were aged 18–25 (*n*-384), 36% 26–35 (*n*-651), and 43% 35–45 (*n* = 788). A considerable proportion of the cohort was classified as overweight (692 individuals, 38%) or obese (419, 23%). There were 422 current smokers (23%) and 58 individuals with family histories of GC(3%). Regarding diet, the consumption of high-salt foods and red/processed meats was reported by 55% (*n* = 1012) and 68% (*n* = 1232) of participants, respectively, while fruit and vegetable intake was reported by 34% (*n* = 623) of participants. Regarding chronic health conditions, a considerable proportion, particularly in terms of the participants’ ages, had DM (21%, *n* = 386) and 26% (*n* = 474) had autoimmune diseases. In particular DM cases included a mixture oftype 1 and 2, and particularly younger onset type 2 diabetes cases.

The overall prevalence of *H. pylori* infection was 78.0% (*n* = 1422). *H. pylori* infection differed significantly across age groups (*p* < 0.001), being higher in the older age category (36–45 years) compared to the younger groups. No statistically significant difference in *H. pylori* infection was observed between males and females (*p* = 0.371). Overall, *H. pylori* was successfully eradicated in 1422 subjects,1299(86%) with non-bismuth quadrable treatment and probiotics, and in123 (14%) with bismuth quadruple treatment and probiotics as a second-line treatment.

**Figure 2:**
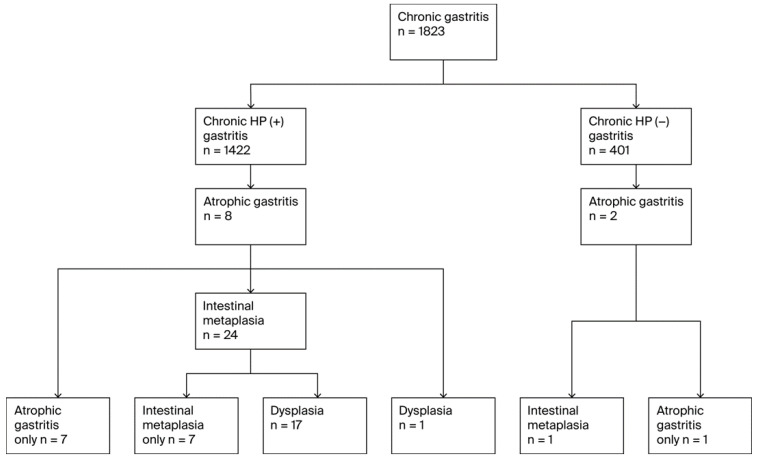
Flow diagram of PGL diagnoses.

A total of 34 unique individuals were diagnosed with at least one premalignant gastric lesion (PGL), resulting in an overall PGL prevalence of 1.9% (*n* = 34). PGL progression followed the Correa cascade (Figure 2). All 34 patients with PGLs had atrophic gastritis (AG),11 of whom were diagnosed with pernicious anemia. Among these, 25 (73.5% of AG cases) progressed to GIM and 18 (52.9% of AG cases and 72.0% of GIM cases) further developed dysplasia. Thirty-two of the AG cases (94.1%) occurred in *H. pylori*-positive participants, and two occurred in *H. pylori*-negative participants. Of the 25 GIM cases, 24 (96.0%) were *H. pylori* positive individuals (all of whom had preceding AG), while one was *H. pylori*-negative(but also had preceding AG). Five patients also had pernicious anemia. All 18 cases of dysplasia occurred in *H. pylori*-positive individuals; 17 of them also had GIM, while one case of dysplasia that developed from AG without intervening GIM was noted. Six dysplasia cases had pernicious anemia including five patients who progressed from GIM and one who did not have GIM but progressed from AG to dysplasia.

**Figure 3:**
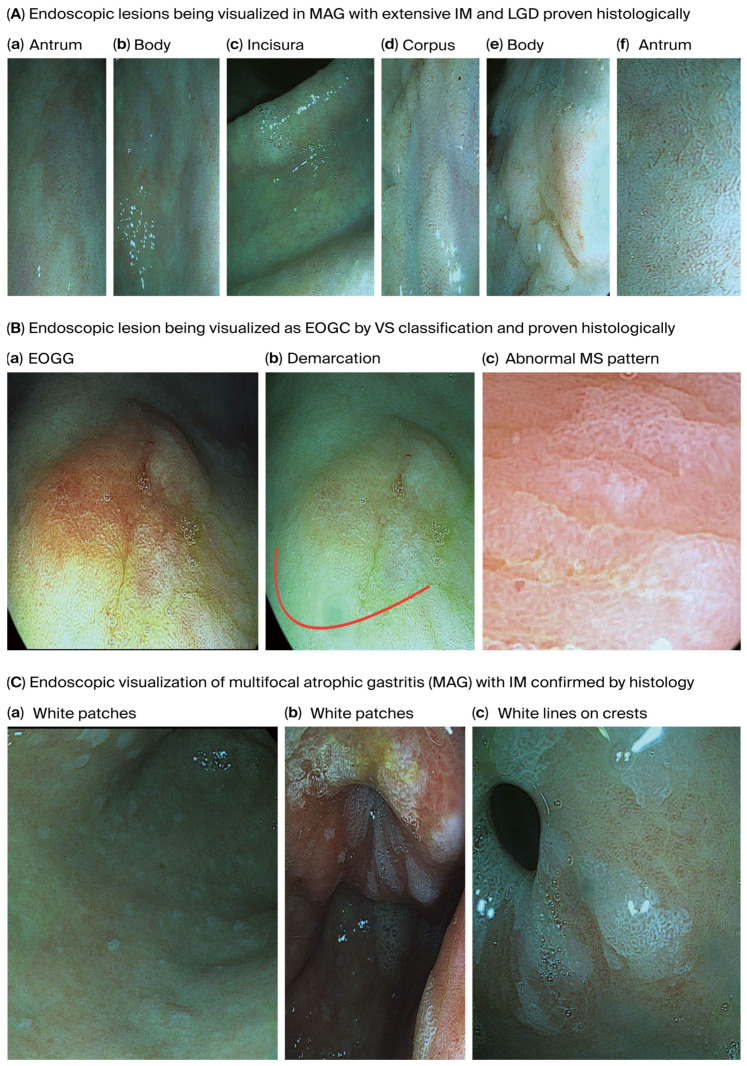
Endoscopically visualized PGLs and EOGC in young adults were confirmed histologically using a high-definition iScan-2, an image-enhancing endoscopy technology from Pentax, Japan. (**A**). **Endoscopic lesions visualized on MAG with extensive IM and LGD**; (**a**) antrum, (**b**) body: some regions in the antrum and body appeared erythematous due to coexisting chronic inflammation, (**c**) incisura and (**d**) corpus: the gastric mucosa appears pale and thinned, especially in the corpus due to glandular atrophy, while the gastric pit pattern is irregular or absent and there is increasing visualization of submucosal blood vessels due to epithelial thinning in the incisura and corpus, (**d**) corpus and (**e**) body: irregular margins and depressions were observed, and atrophic areas are interspersed with nodular or depressed lesions. (**f**) antrum: IM is visible with patchy atrophy, with areas with a mosaic pattern and whitish changes suggesting IM in the antrum, (**d**) corpus and (**f**) antrum: variable vascular density is observed within the gastric mucosa resulting from metaplastic processes present in the antrum and corpus and white lines on the crests of the epithelial surfaces, (**d**) corpus and (**e**) body: the gastric mucosa in the IM displays a tubule-villous or irregular configuration in the body and corpus, reflecting IM, low-grade dysplasia is displayed by depressed morphology lesions present in the corpus and body around the surrounding mucosa, (**a**) antrum, (**c**) incisura and body (**b**): the mucosal surface of the body and corpus displays uneven surface nodularity indicating underlying dysplasia. The surface erythema in the antrum/incisura and body (**a**,**c**,**b**) may be associated with dysplastic changes and low-grade dysplasia, the prominent submucosal veins became more conspicuous in the incisura, indicating AG. (**B**). **Endoscopic lesion visualized as EOGC by VS classification**; (**a**) EOGC and (**b**) demarcation red colour line: an elevated flat lesion with erosion was visualized in the transitional region between the antrum and body of the stomach, (**c**) abnormal MS pattern: irregular microsurface patterns (MSs) with disrupted and irregular epithelial surface structures reflecting abnormal cellular architecture, he presence of a white opaque substance (WOS) within the epithelium is commonly associated with neoplastic lesions and irregular almost absent microvascular pattern (AV), absent microvessel (MV) patterns or irregular distorted patterns within the lesion. A demarcation red colour line with a clear boundary between the cancerous and non-cancerous mucosa can be visualized due to abrupt changes in the mucosal pattern. (**C**). **Endoscopic visualization of multifocal atrophic gastritis (MAG) with IM**. (**a**) body and (**b**) antrum: sticky adherent dense mucus, a white mucus layer that adheres firmly to the mucosa and is resistant to removal with water irrigation, indicates MAG, (**c**) antrum: white lines on the crests of epithelial surfaces indicating IM. Abbreviations: MAG—multifocal atrophic gastritis; MS—microsurface pattern; MV—microvessel pattern; VS—microvascular surface vessels plus surface; WOS—white opaque substance.

Figure 3 shows endoscopically visualized PGLs and EOGC at an early stage using a zoom (magnification) high-definition virtual ChromoendoscopyiScan-2, image enhancing technology from Pentax, Japan, which was confirmed histologically.

The analysis of risk factors associated with PGLs, as displayed in Table 2, showed that *H. pylori* infection was significantly more prevalent among individuals with PGLs compared to those without (*p* = 0.022). Increasing age was also a significant factor (*p* < 0.001), particularly in the age group of 26–35 years. A family history of GC was strongly associated with the presence of PGLs (*p* < 0.001). Among dietary factors, no consumption of fruits, vegetables, or unprocessed wheat products was significantly associated with PGLs (*p* < 0.001), as was obesity. Smoking was also a risk factor, while gender, autoimmune conditions and consumption of red/processed meats and salty preserved foods did not show a statistically significant association with PGLs in this cohort.

The histopathological grading of PGLs using the OLGA and OLGIM classification systems is shown in Table 3. For atrophic gastritis (*n* = 34), most cases were classified as OLGA stage II or III, indicating moderate to severe stages of atrophy based on the location of the stomach. Five cases classified as OLGA grade III also had extensive involvement, indicating high cancer risk. For GIM (*n* = 25), the majority were classified as OLGIM Stage II or III, indicating moderate to severe stages of IM, with a smaller number being classified as OLGIM Stage I. Five cases classified as OLGIM grade III also had extensive involvement, indicating high cancer risk.

Fifteen participants (0.8% of the total cohort) were diagnosed with EOGC. Twelve of these cases were adenocarcinomas (EOGC), all of which arose from the 18 patients with dysplasia within the PGL cohort, representing progression from these advanced lesions. The remaining three GC cases included two MALT lymphomas and one GIST, whose development was distinct from the PGL–adenocarcinoma sequence. All patients with EOGC were *H. pylori*-positive and five had pernicious anemia. When examining risk factors (Table 4), age group (particularly the 36–45 group) and BMI > 30 kg/m^2^ were significantly associated with EOGC. Additionally, *H. pylori* infection was significantly more common in patients with EOGC compared to those without cancer (*p* < 0.001), as was a family history of gastric cancer (*p* < 0.001). EBV infection, DM, and smoking were also significantly associated with EOGC, while gender, diet, and autoimmune conditions were not associated with EOGC.

Binary logistic regression analysis was performed to identify the possible risk factors associated with PGLs (Table 5) and EOGC (Table 6) in young adults (available in the Supplementary Materials), *H. pylori* status, EBV, demographic factors, lifestyle habits (smoking, BMI, and diet), and relevant comorbidities such as diabetes mellitus were used as independent variables in the binary logistic regression. Similar results shown by binary logistic regression, significant risk factors (*p* < 0.05) for PGLs were older age, obesity (BMI > 30), *H. pylori* infection, EBV infection, DM, autoimmune atrophic gastritis (pernicious anemia), pernicious anemia associated with autoimmune diseases, current smoking, and family history of GC. Significant risk factors were the common ones for EOGC, including histopathology and male gender (males had a lower risk but this was borderline significant). However, the study had a small cohort size and sparse events, i.e., rare outcomes in young adults, and, especially in younger age groups, high variability in data and model instability meant there is some uncertainty about the magnitude of the effect of the possible risk factors associated with PGLs and EOGC.

## 4. Discussion

This observational cohort study with a longitudinal design provides important insights into the prevalence of PGLs and EOGC among symptomatic young adults in central Saudi Arabia, a region characterized by a high prevalence of *Helicobacter pylori* infection but an overall low incidence of GC [27,28,29]. Our key findings revealed a notable prevalence of *H. pylori* infection (78.0%), a PGL prevalence of 1.9% (encompassing AG, GIM, and dysplasia), and an EOGC (adenocarcinoma) prevalence of 0.7% in this cohort. Significant risk factors for PGLs included *H. pylori* infection, increasing age, a family history of GC, a lack of regular fruit/vegetable/unprocessed wheat consumption, obesity, and smoking. For EOGC-adenocarcinoma, significant associations were found with older age within the cohort (36–45 years), obesity, *H. pylori* infection, a family history of GC, EBV infection, DM, and smoking.

The high prevalence of *H. pylori* infection observed in our young adult cohort is consistent with previous reports from Saudi Arabia [29,30,31] and underscores its endemicity. The significant increase in *H. pylori* prevalence with age, even within this relatively young demographic, suggests ongoing acquisition or higher cumulative exposure in slightly older individuals. This high burden of *H. pylori* is a critical backdrop for understanding the development of PGLs and EOGC, as the infection is a primary driver for both [1,11].

The overall PGL prevalence of 1.9% in symptomatic young adults was a significant finding. While global adult prevalence data for PGLs exist [21,22], data specifically for young adult cohorts, particularly from the Middle East, are scarce. The observed progression from AG (100% of PGL cases) to GIM (73.5% of AG) and then to dysplasia (52.9% of AG and 72.0% of GIM) aligns with the Correa cascade of gastric carcinogenesis [12,15]. The observation that 94.1% of AG cases and all dysplasia cases occurred in *H. pylori*-positive individuals is in agreement with the well-established pivotal role of this infection in initiating and promoting precancerous changes. The identification of PGLs in this young, symptomatic population highlights that the carcinogenic process can be initiated early in life, warranting vigilance.

The histopathological staging by the OLGA and OLGIM systems revealed that most AG and GIM cases were classified as stage II or III. These stages are associated with a significantly increased risk of GC development [36,37,38], underscoring the need for endoscopic surveillance in such individuals, even if young, as per guidelines like MAPS III [12]. The use of high-definition chromoendoscopy was instrumental in visualizing and targeting these subtle mucosal changes, emphasizing the importance of advanced endoscopic techniques in detecting PGLs and early EOGC.

Our analysis of the factors associated with PGLs identified several established and emerging correlates. The strong association between *H. pylori* infection, increasing age, and family history of GC is well-documented [1,12,13,14,15,16]. The link between a diet lacking regular fruit, vegetable, and unprocessed wheat product intake and PGLs aligns with concerns about “Western” dietary patterns increasing GC risk [1,11]. Importantly, obesity and smoking are well documented risk factors for PGLs. Obesity is increasingly recognized as a risk factor for various cancers, including GC [41,42], and our findings suggest its relevance even in the early stages of gastric carcinogenesis in young adults. Smoking is a well-established risk factor for GC [19,20], and its association with PGLs in our young cohort was consistent.

The EOGC-adenocarcinoma prevalence of 0.7% in our study, although seemingly low, is concerning given the young age of the cohort. All 12 adenocarcinoma cases arose from the 18 patients with dysplasia, emphasizing direct progression from advanced PGLs to invasive cancer and the critical importance of identifying and managing dysplasia. This finding supports international guidelines that recommend surveillance for high-risk PGLs [12,13,14,15,16].

For EOGC-adenocarcinoma, the risk factor profile showed some overlapping but also distinct features. All EOGC-adenocarcinoma patients were *H. pylori*-positive, and the infection was significantly more common compared to those without cancer. This was expected, considering that *H. pylori* infection is an important risk factor for GC [1,41]. Older age within this EOGC cohort and a family history of GC were strongly correlated, consistent with the existing EOGC literature [1,10]. Obesity has also been associated with EOGC-adenocarcinoma, suggesting that its role may be potentiated in the later stages of carcinogenesis or that it contributes through systemic effects promoting cancer development [42,43]. Smoking was also associated with EOGC. The association between EBV infection and EOGC in our study is noteworthy, as EBV is implicated in approximately 10% of GCs globally [40,44], and its role in EOGC warrants further exploration. DM was also significantly associated with an EOGC. The link between DM and GC risk has been reported in meta-analyses and is possibly mediated by hyperinsulinemia, inflammation, or oxidative stress [45,46]. Interestingly, while a diet low in fruits/vegetables/unprocessed wheat was associated with PGLs, no such correlation was found with EOGC-adenocarcinoma, possibly because of the small sample size.

The strengths of our study include its prospective design, the inclusion of a relatively large cohort of symptomatic young adults from multiple centers in central Saudi Arabia, systematic endoscopic evaluation using high-definition chromoendoscopy, and standardized histopathological assessment by an experienced pathologist according to the Sydney system and OLGA/OLGIM staging.

However, this study has several limitations. As a hospital-based study of symptomatic individuals, its findings are not generalizable to the asymptomatic young adult population in Saudi Arabia; PGL and EOGC prevalence are most likely overestimated compared to the general population. The number of EOGC cases was relatively small, which may have limited the statistical power for disease correlates. Dietary and lifestyle data were collected using a questionnaire, which was subject to recall bias. Finally, we did not assess *H. pylori* virulence factors (e.g., CagA and VacA) or specific host genetic polymorphisms beyond family history, which could further modulate the risk. Finally, the “autoimmune conditions” category in the tables was broad in the EOGC risk analysis; specific investigation for pernicious anemia through serology was only performed in select cases, though no overt cases were identified through baseline characteristics or targeted testing based on histological indications. Another limitation was that although it is recommended to use high-quality endoscopy systems such as image enhancement to optimize mucosal visibility, additionally, photographic documentation and a standardized biopsy protocol should be followed for proper mucosal staging [16,47]. In our study, microphotographs were not available. Staging was based on score sheets using the OLGA/OLGIM systems and histological conformation of endoscopic lesions on histological reports. Finally, logistic regression analysis was performed to identify possible factors associated with PGLs (*H. pylori* status, EBV, demographic factors, and lifestyle factors (smoking, BMI, and diet)), relevant comorbidities such as diabetes mellitus applied to the small cohort size, and rare outcomes in young adults and especially in younger age groups, so the magnitude of the effect of the possible associated risk factors with PGLs and EOGC may be less accurate. The high variability in data and model instability also creates some uncertainty around the magnitude of the effect of the possible risk factors associated with PGLs and EOGC. Future prospective studies with larger and more robust cohorts aimed at estimating the factors associated with PGLs and EOGC more precisely are needed.

Clinically, our findings suggest a need for increased awareness and a lower threshold for endoscopic evaluation in symptomatic young adults in Saudi Arabia, particularly those with risk factors such as a family history of GC, obesity, smoking, DM, or evidence of *H. pylori* or EBV infection. The identification of high-risk PGLs (OLGA/OLGIM III and dysplasia) necessitates surveillance to detect EOGC at an earlier, more curable stage [12,16].Future research should focus on larger, population-based studies to determine the true prevalence of PGLs and EOGC in asymptomatic young Saudis. Longitudinal studies are needed to better understand the natural history and progression rates of PGLs in this younger population. Further investigation into the interplay between *H. pylori* virulence, host genetics, EBV, and specific dietary and metabolic factors (such as detailed components of obesity and DM) is crucial for refining risk stratification and developing targeted prevention or screening strategies for EOGC in this high *H. pylori* prevalence setting.

In conclusion, this study demonstrates that PGLs and EOGC are not uncommon findings in symptomatic young adults in central Saudi Arabia and are strongly associated with *H. pylori* infection, along with a constellation of demographic, lifestyle, and comorbidity risk factors including obesity, smoking, family history, EBV, and DM. These findings underscore the importance of early detection and risk factor management in this vulnerable population.

## Figures and Tables

**Table 1:** Summary of baseline data of study participants (N = 1823).

Factor	N	%
**Gender**		
Male	981	54
Female	842	46
**Age(mean, SD): 36 ± 11.3**		
**Age groups**		
18–25	384	21
26–35	651	36
36–45	788	43
**BMI**		
Normal (<25 kg/m^2^)	712	39
Overweight (25–30 kg/m^2^)	692	38
Obesity (>30 kg/m^2^)	419	23
**Diabetes Mellitus**		
Yes	386	21
No	1437	79
**Autoimmune conditions**		
Yes	474	26
No	1349	74
**Smoking status (cigarettes, vape, shisha)**		
Non-smoker	1112	61
Former smoker	289	16
Current smoker	422	23
**Diet**		
Fresh fruits, vegetables, unprocessed wheat products	623	34
Animal products, hot spices, canned and fermented foods	1232	68
Nutritional salty preserved products	1012	55
**Family history with GC**		
Yes	58	3
No	1765	97

**Table 2:** Summary of participants’ variables with and without PGLs. Data are presented as numbers (%). The table includes 12 cases that had PGLs but were also diagnosed with EOGC.

Factor	With PGLs	Without PGLs	*p* Value
	(*n* = 34)	(*n* = 1789)	
**Gender**			
Male	13 (1.33)	968 (98.67)	0.066
Female	21 (2.50)	821(97.600)	
**Age groups**			
18–25	1 (0.26)	383 (99.74)	0.001
26–35	6 (0.9.3)	645 (99.07)	
36–45	27 (3.40)	761 (96.60)	
**BMI**			
Normal (<25 kg/m^2^)	3 (0.42)	709 (99.58)	<0.001
Overweight (25–30 kg/m^2^)	5 (0.72)	687 (99.28)	
Obesity (>30 kg/m^2^)	26 (6.20)	393 (93.80)	
***H. pylori* Infection**			
Yes	32 (2.25)	1390 (97.75)	0.022
No	2 (0.45)	399(99.55)	
**EBV**			
Yes	20 (9.62)	188 (90.38)	<0.001
No	14 (0.87)	1601 (99.13)	
**Diabetes Mellitus**			
Yes	13 (3.37)	373 (96.63)	0.013
No	21 (1.46)	1416 (98.54)	
**Autoimmune conditions**			
Yes	5 (1.05)	469 (98.95)	0.129
No	29 (2.15)	1320 (97.85)	
**Smoking status (cigarettes, vape, shisha)**			
Non-smoker	4 (0.36)	1108 (99.64)	<0.001
Former smoker	11 (3.81)	278 (96.19)	
Current smoker	19 (4.5%)	403 (95.55)	
**Diet**			
Fresh fruits, vegetables, unprocessed wheat products			<0.001
Yes	1 (0.16)	622 (99.84)	
No	33 (2.75)	1167 (97.25)	
Animal products, hot spices, canned and fermented foods			
Yes	18 (1.46)	1214 (98.54)	0.065
No	16 (2.70)	575 (97.3)	
Nutritional salty preserved products			0.177
Yes	15 (1.48)	997 (98.52)	
No	19 (2.34)	792 (97.66)	
**Family history with GC**			
Yes	11 (19.96)	47 (80.04)	<0.001
No	23 (1.30)	1742 (98.70)	

**Table 3:** OLGA and OLGIM classification systems.

OLGA Staging System		Antrum + Incisura	Corpus	Extensive
N = 34		20	14	11
0	0	0	0	0
I	13	8	5	2
II	9	5	4	4
III	12	7	5	5
**OLGIM staging system**				
**N = 25**		**14**	**11**	**9**
0	0	0	0	0
I	5	3	2	0
II	9	5	4	4
III	11	6	5	5

**Table 4:** Summary of participants’ variables with and without EOGC. Data are presented as number (%). All cases of EOGC were from the PGLs cohort.

Factor	With EOGC (*n* = 12) *	Without EOGC (*n* = 1811)	*p* Value
**Gender**			
Male	4 (0.41)	977 (99.59)	0.153
Female	8 (0.95)	834 (99.05)	
**Age groups**			
18–25	1 (0.26)	383 (99.74)	0.018
26–35	1 (0.15)	650 (99.85)	
36–45	10 (1.26)	778 (98.74)	
**BMI**			
Normal (<25 kg/m^2^)	1 (0.14)	711 (99.86)	<0.001
Overweight (25–30 kg/m^2^)	2 (0.29)	690 (99.71)	
Obesity (>30 kg/m^2^)	9 (2.15)	410 (97.85)	
**EBV**			
Yes	4 (1.92)	204 (98.08)	0.016
No	8 (0.40)	1607 (99.60)	
**Diabetes Mellitus**			
Yes	9 (2.33)	377 (97.77)	0.001
No	3 (0.21)	1434(99.79)	
**Autoimmune conditions**			
Yes	4 (0.84)	470 (99.16)	0.56
No	8 (0.59)	1341(99.41)	
**Smoking status (cigarettes, vape, shisha)**			
Non-smoker	1 (0.08)	1111(99.92)	<0.001
Former smoker	2 (0.69)	287 (99.31)	
Current smoker	9 (2.10)	413 (97.90)	
**Diet**			
Fresh fruits, vegetables, unprocessed wheat products			
Yes	2 (0.32)	621 (99.68)	0.058
No	10 (0.83)	1190 (99.17)	
Animal products, hot spices, canned and fermented foods			
Yes	6 (0.49)	1226 (99.51)	0.191
No	6 (1.01)	585 (98.99)	
Nutritional salty preserved products			
Yes	5 (0.49)	1007 (99.51)	0.332
No	7 (0.86)	804 (99.14)	
**Family history with GC**			
Yes	5 (9.43)	53 (90.57)	<0.001
No	7 (0.40)	1758 (99.60)	

* Two MALT cases and one GIST case were not included in this table.

**Table 5:** Risk factors associated with PGLs, examined by binary logistic regression analysis.

Factor	OR	95% CI	*p*
**Gender**			
Male	Ref.		
Female	1.11	1.031–1.04	0.981
**Age**			
18–25	Ref.		
26–35	2.34	1.51–3.62	0.001
36–45	4.23	1.36–13.1	0.004
**BMI**			
Normal (<25 kg/m^2^)	Ref.		
Overweight (25–20 kg/m^2^)	0.89	0.5–1.5	0.682
Obesity (>30 kg/m^2^)	1.98	1.18–3.32	0.009
**Diabetes Mellitus**			
No	Ref.		
Yes	2.24	1.11–4.52	0.025
**Autoimmune atrophic gastritis, pernicious anemia**			
No	Ref		
Yes	3.32	2.31–4.77	0.001
**Pernicious anemia and autoimmune conditions**			
No	Ref.		
Yes	4.87	2.15–11.03	0.001
**Autoimmune conditions**			
No	Ref.		
Yes	0.47	0.18–1.23	0.124
**Smoking status**			
Non-smoker	Ref.		
Former smoker	0.61	0.21–1.77	0.9
Current smoker	1.78	1.45–2.18	0.001
**Diet**			
Fresh fruits, vegetables, unprocessed wheat products	Ref.		
Animal products, hot spices, canned and fermented foods	0.68	0.36–1.29	0.234
Nutritional salty preserved products	0.64	0.33–1.23	0.180
**Family history with GC**			
No	Ref.		
Yes	1.70	1.41–2.49	<0.001
***Helicobacter pylori* infection**			
No	Ref.		
Yes	2.1	1.60–2.75	<0.001
**EBV**			
No	Ref.		
Yes	1.42	1.09–1.85	0.009

Abbreviations: OR (odds ratio), 95% CI (95% confidential interval).

**Table 6:** Risk factors associated with EOGC, examined by binary logistic regression analysis.

Factor	OR	95% CI	*p*
**Gender**			
Female	Ref.		
Male	0.45	0.21–0.98	0.045
**Age**			
18–25	Ref.		
26–35	0.98	0.12–7.35	0.990
36–45	1.60	1.11–2.30	0.011
**BMI**			
Normal (<25 kg/m^2^)	Ref.		
Overweight (25–20 kg/m^2^)	0.98	0.83–1.15	0.68
Obesity (>30 kg/m^2^)	1.81	1.16–2.82	0.001
**Diabetes Mellitus**			
No	Ref.		
Yes	1.77	1.14–3.01	0.021
**Autoimmune atrophic gastritis, pernicious anemia**			
No	Ref		
Yes	4.41	2.62–7.42	<0.001
**Pernicious anemia and autoimmune conditions**			
No	Ref.		
Yes	2.88	1.19–5.88	0.001
**Autoimmune conditions**			
No	Ref.		
Yes	1.09	0.89–1.33	>0.05
**Smoking status**			
Non-smoker	Ref.		
Former smoker	0.91	0.78–1.06	0.179
Current smoker	2.38	1.56–3.63	<0.001
**Diet**			
Fresh fruits, vegetables, unprocessed wheat products	Ref.		
Animal products, hot spices, canned and fermented foods	0.39	0.14–1.09	0.072
Nutritional salty preserved products	0.36	0.12–1.08	0.067
**Histology**			
Chronic HP gastritis	Ref.		
Atrophic gastritis	2.67	1.90–3.75	<0.001
Intestinal metaplasia	3.61	2.09–6.23	<0.001
Dysplasia	12.14	5.98–24.64	<0.001
**Family history with GC**			
No	Ref.		
Yes	2.86	1.22–5.17	0.001
***Helicobacter pylori* infection**			
No	Ref.		
Yes	3.06	2.01–4.66	<0.001
**EBV**			
No	Ref.		
Yes	1.81	1.31–2.50	0.03

Abbreviations: OR (odds ratio), 95% CI (95% confidential interval).

## Data Availability

The data are available from the corresponding author upon reasonable request.

## Supplementary Materials

The following supporting information can be downloaded at: https://www.mdpi.com/article/10.3390/curroncol32080428/s1, File S1. questionnaire to collect sociodemographic data and possible factors associated with gastric cancer (GC).
