# Supplementary material for: A Longitudinal Study of Premalignant Gastric Lesions and Early Onset Gastric Cancer Among Young Adults in Central Saudi Arabia
Source: Curr Oncol. 2025 Jul 30;32(8):428. doi: 10.3390/curroncol32080428 (PMC12384965; doi:10.3390/curroncol32080428)
Supplement: Supplementary file 1 [file curroncol-32-00428-s001.zip › curroncol-3726870-supplementary.pdf]

A Longitudinal Study of Premalignant Gastric Lesions and Early Onset Gastric Cancer Among Young Adults in Central Saudi Arabia

Supplementary materials: Questionnaire to collect sociodemographic data and possible factors associated with gastric cancer (GC).

Survey Questionnaire: Factors that possibly associated with GC included in the questionnaire were age, obesity (body mass index (BMI) > 30), smoking, a diet of salty preserved foods, income, education and a family history of GC. The questions and answers were collected during history taking by the visit of the eligible participant in the study at the family medicine outpatient clinic.

SOCIODEMOGRAPHIC AND RISK FACTORS FOR EARLY ONSET GASTRIC CANCER AND

PREMALIGNANT LESIONS QUESTIONNAIRE

Only for those who were positive in a high-sensitivity guaiac fecal occult blood test (HSgFOBT+) and had negative results in colonoscopy and offered to undergo upper GI endoscopy.

Pt. No. ☐☐☐☐☐☐☐☐ *Seq. No. ☐☐

**Step No. Date

☐☐☐☐☐☐☐☐☐

The questions and answers

1. Age:

2. Gender: Female……..Male……Other…….

3. What is your nationality? Saudi…Non-Saudi… Ethnicity? Arab…… Afro-Arab………other….

4. Weight (kg)

5. Height (cm)

6. BMI:

A. Classify accordingly the following options:

a.	Normal (18.5–25 kg/m²)……….
b.	Overweight (25–30 kg/m²)…….
c.	Obese (>30 kg/m²)………………

7. Are you being overweight?

8. Marital status:

- Single………..
- Married……..
- Divorced…....
- Widowed…….

9. Do you have unexplained weight loss? ☐

5. Have you ever undergone upper GI endoscopy? ☐

6. Do you have any upper GI endoscopy symptoms? ☐

7. Common Warning Signs and Symptoms of Gastric Cancer ☐

Report one or more of these symptoms if you have:

A. Early Symptoms (often vague and non-specific):

Indigestion or heartburn that doesn't go away ☐

Feeling bloated after eating small amounts ☐

Loss of appetite ☐

Mild nausea ☐

Discomfort or pain in the upper abdomen ☐

Unexplained fatigue ☐

B. Late Symptoms (more specific and serious):

Unintentional weight loss ☐

Persistent vomiting, especially if it contains blood ☐

Difficulty swallowing (dysphagia) – usually suggests cancer in upper stomach ☐

Visible blood in vomit (hematemesis) or black, tarry stools (melena) ☐

Persistent abdominal pain, especially in the epigastric area ☐

Anemia-related symptoms (pale skin, dizziness, breathlessness) ☐

Feeling full quickly (early satiety) ☐

Swelling or fluid in the abdomen (ascites – seen in advanced stages) ☐

C. Red Flag Symptoms (require urgent evaluation)☐

Weight loss with persistent upper GI symptoms ☐

Progressive difficulty swallowing ☐

Vomiting blood or black stools☐

Family history of gastric cancer + new GI symptoms☐

Age >40–50 years with new-onset dyspepsia☐

8. Responses to the Prompted Items of Gastric Cancer Warning Signs and Symptoms (2nd round)

a. Do you have black stools from back passage?
b. Do you have persistent pain in abdomen-epigastric pain or stomach burning, bloating, early satiety?
c. Do you have a change of bowel habits (diarrhea, constipation) over weeks
d. Do you give persistent vomiting over weeks?

e. Do you feel a Lump in your abdomen?

f. Do you have occasionally retrosternal pain and or regurgitation of food?

g. Do you have chest pain?

h. Do you have difficulty in swallowing or pain?

i. Do you feel Is Tiredness/anaemia?

j. Unintentional weight loss

k. Persistent vomiting, especially if it contains blood

l. Difficulty swallowing (dysphagia) – usually suggests cancer in upper stomach

m. Visible blood in vomit (hematemesis) or black, tarry stools (melena)

n. Persistent abdominal pain, especially in the epigastric area

o. Anemia-related symptoms (pale skin, dizziness, breathlessness)

p. Feeling full quickly (early satiety)

q. Swelling or fluid in the abdomen (ascites – seen in advanced stages)

9. Have you ever tested for Helicobacter Pylori infection ? If yes please answer

    1. POSITIVE? ☐

       RECEIVED TREATMENT? ☐

    2. NEGATIVE? ☐

10. Medical history

a. Do you have history of Inflammatory bowel disease?

b. Do you have history of gastric cancer?

c. Do you have history of Barrett's exophagous

d. Do you have history of familial adenomatous polypoposis or Lynch?

e. Any surgery if the past of the GI tract?

f. Do you have pernicious anemia?

g. Do you have one or more autoimmune diseases?

h. Are you diabetic?

11.. Life style factors

A. Smoking status

A. Do you currently smoke?

• Yes • No

B. (only for current smokers) Based on your opinion/knowledge, please report which of the following products you are using weekly

• Tobacco cigarettes • Snus

• Electronic cigarettes

• Nicotine replacement therapies

• Shisha or Hookah

• Oral smoking cessation medications (varenicline, bupropion)

C. Have you ever smoked in the past?

• Yes • No

D. Please classify the participant accordingly to the following options:

a. Smoking status

b. Never smoked

c. Former smoker

d. Current smoker

12. Do you have a close relative with Gastric Cancer

13. Family history of GC

a. Yes

b. No

14. Dietary Factors

How many times have practiced physical activity (at least 30 minutes)?

- One time per week………………………………
- 3-1 times per week………………………………
- More than 3 times per week……………………
- None……………………………………………….

Diet

A. What are the foods you eat weekly? (Multiple options are available)

- Do you eat less than 5 portions of fruit and vegetables?
- Are you having a diet low in fiber daily?
- Do you do frequent consumption of deep fried foods?
- Do you eat red meat or processed meat more than once a day?
- Apple, orange, grapefruit, date, grape, banana, other fruits not mentioned
- Tomato, Cucumber, onion, carrot, garlic, cabbage, eggplant, other vegetables not mentioned
- Beans, Lentils, chickpeas, Lupine
- Coffee, tea
- Other

B. How many times per week you eat fresh fruits, vegetables, unprocessed wheat products?

- 1-3 times per week
- 4-7 times per weeks
- More than 7 times per week
- None

C. Do you eat fast food on a regular basis?

Yes…

No…

D. (only for those answered "yes"). How many times you eat on fast food on a weekly basis on average?...

E. Based on your opinion/knowledge, please score the health risk of each of the following products from 1 (lowest risk) to 10 (highest risk).

a. Fresh fruits, vegetables, unprocessed wheat products………………………………

b. Animal products, hot spices, canned and fermented foods…………………………

c. Nutritional salty preserved products…………………………………………………..

F. (only for users of nutritional salty preserved products) How many times per week?

- 1-3 times per week
- 4-7 times per weeks
- More than 7 times per week
- None

G. (only for users of salty preserved products) Based on your opinion/knowledge, please report which of the following products you are using

1. Salted fish (such as dried and salted cod or salted herring)
2. Salt-cured meat (such as beef bacon)
3. Pickles, cheeses, fermented beef sausages
4. vegetables such as runner bens and cabbage
5. other salted products……………….

E. (only for users of animal products, hot spices, canned and fermented foods) How many times per week?

- 1-3 times per week
- 4-7 times per weeks
- More than 7 times per week
- None

Thank you very much for completing this questionnaire.
